# Supplementary material for: Statin Use in Relation to COVID-19 and Other Respiratory Infections: Muscle and Other Considerations
Source: J Clin Med. 2023 Jul 13;12(14):4659. doi: 10.3390/jcm12144659 (PMC10380486; doi:10.3390/jcm12144659)
Supplement: Supplementary file 1 [file jcm-12-04659-s001.zip › jcm-2465845-supplementary.pdf]

Statin Use in Relation to COVID-19 and Other Respiratory Infections: Muscle and Other Considerations

Supplement I: Illustrations of Endorsement of Statin Use, and Statements Appearing to Support Statin Use for Acute COVID-19

I. Multiple Early Endorsements of Statin Use in Treatment of COVID-19

a. Massachusetts General Hospital (MGH) issued an unauthored, undated position piece entitled "Rationale for Consideration of Statins for COVID-19 Patients" that suggests the off-label use of high-dose statins in patients infected with COVID-19 [1]; this, for some critical months following the first recognition of the COVID-19 pandemic, sustained the top spot on a Google search on "COVID-19 and statins" (Supplement Figure 1).

b. The Eastern Virginia Medical School (EVMS) Medical Group [2] included an "optional" recommendation for statin use, among "additional treatment approaches," in their "COVID-19 Management Protocol".

c. A "correspondence" in the European Heart Journal nominally recommended phase III clinical trials of statins, but de facto promoted statin use in the interim: "In conclusion, statins are low-cost, extensively tested, well-tolerated drugs that are less likely to be affected by a shortage in a health crisis such as the current COVID-19 pandemic, even in low-income countries, where treatment with more expensive drugs may not be implemented. Adjuvant treatment and continuation of pre-existing statin therapy could improve the clinical course of patients with COVID-19, either by their immunomodulatory action or by preventing cardiovascular damage" [3]. More explicitly, it was said "In this paper we support the rationale for the use of statins, a class of drugs

with widespread availability and an optimal tolerability profile, as an add-on treatment for COVID-19 patients, on the basis of their known immunomodulatory properties" [3]. It is noteworthy that this article described "an optimal tolerability profile" for statins [3]. Meanwhile, in keeping with this sanguine view of statin safety, the MGH document's table had a column entitled "Key Toxicities," which was empty for the statin entry, implying apparent absence of potential for toxicity (Table S1).

Supplement Figure S1. Top "COVID-19 and statins" Google Search Result and Recommendations in Associated Document

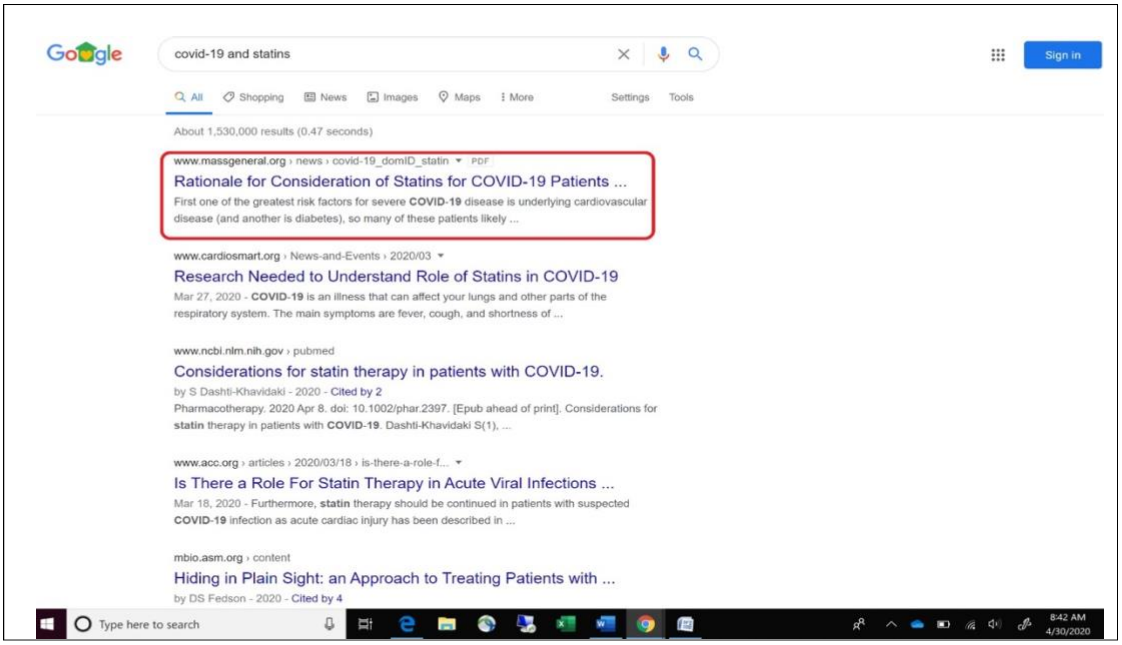

The PDF [1] generated by following the link shown above states: "Recommendation: Continue statins if already prescribed. If no contraindication, and for those who have a guideline indication for a statin, consider starting either atorvastatin 40 mg daily or rosuvastatin 20 mg daily. When major drug-drug interactions with atorvastatin or rosuvastatin are expected, pitavastatin 2 mg daily (or pravastatin 80 mg daily if pitavastatin not available) should be considered" [1].

In sentence two, the comma preceding "and," and the wording "and for those" rather than "for those," suggest that members of either group should receive statins.

This specific search was performed on 4/30/2020; however, the top result remained stable through at least 6/10/2020 as well as for weeks prior to the 4/30/2020 search. Thus, that number one search result had significant opportunity to influence physician behavior at a critical time. The recommendations are not accessed by following the above link, but are available through massgeneral.org. Another (dated) version (Version 3.0, 5/1/2020, 6:00PM; Tables S1 & S2) advised continuing existing statins even with new LFT abnormalities, initiating statins if there is a pre-existing primary indication. Although otherwise, for statin-naïve patients, *that* document stated, "this would be best studied under a clinical trial" and statins "should not be specifically started for COVID-19," and although there have been subsequent revisions, the link shown above has continued to be the top search hit, and continued to direct viewers instead to the recommendation noted herein (as of 6/10/2020).

Table S1. Unauthored MGH recommendations dated 3-17-2020. Table from MGH generated PDF version 1.0 provided by MGH website (lopinavir/ritonavir and Interferon beta-B1 rows were removed later on, in version 2.12).

**MGH Version 1.0**	**Brief Overview**			**3/17/2020**
**Agent**	**Classification**	**Target/Mechanism**	**Dosing**	**Key toxicities**
atorvastatin (Lipitor)	Off-label	Cardioprotection; immunomodulatory	40-80 mg PO daily	
pravastatin (Pravachol)	Off-label	Cardioprotection; immunomodulatory	80 mg PO daily	
**remdesivir**	Investigational	RNA dependent RNA polymerase inhibitor	200 mg IV x 1, then 100 mg IV daily, up to 10 days	Nausea, Vomiting, ALT elevation
hydrochloroquine **(Plaquenil)**	Off-label	Multiple actions: Prevents binding to ACE2, presents transport in endosome, and possibly others 3CLpro (viral protease) inhibitor	400 mg BID x 2 doses, then 200 mg BID for 5 days	QT prolongation
lopinavir/ritonavir (LPV/r or Kaletra)	Off-label	3CLpro (viral protease) inhibitor	400/100 mg BID for up to 10 days	QT prolongation, ALT elevation
Interferon beta-B1 (Betaseron)	Off-label	Immunomodulatory; enhancement of innate and adaptive viral immunity	Dosing for progressive COVID to be determined	Depression, injection site reaction, flue like symptoms
tocilizumab (Actemra)	Off-label	Monoclonal antibody to IL6 receptor/ treats cytokine release syndrome	Dosing for COVID/CRS to be determined	ALT elevation

Table S2. Un-authored MGH recommendations dated 4-20-2020. Table from MGH generated PDF version 2.12 provided by MGH website (favipiravir and sarilumab rows were added in version 2.12).

**MGH Version 2.12**	**Brief Overview**			**4/20/2020**
**Agent**	**Classification**	**Target / Mechanism**	**Dosing**	**Key toxicities**
**remdesivir**	Investigational	RNA dependent RNA polymerase inhibitor	200 mg IV x 1, then 100 mg IV daily, up to 10 days	Nausea, vomiting, ALT elevation
**favipiravir**	Investigational	RNA dependent RNA polymerase inhibitor	Per study protocol	Elevated uric acid
hydrochloroquine **(Plaquenil)**	Off-label	Multiple actions: prevents binding to ACE2, presents transport in endosome, and possibly others	400 mg BID x 2 doses, then 200 mg BID for 5 days	QT prolongation
tocilizuman (Actemra)	Off-label	Monoclonal antibody to IL6 receptor/ treats cytokine release syndrome	Dosing for COVID/CRS to be determined	ALT elevation
sarilumab **(Kevzara)**	Off-label, investigational	Monoclonal antibody to IL-6 receptor	Dosing for COVID/CRS to be determined	ALT elevation
atorvastatin (Lipitor)	Off-label	Cardioprotection; immunomodulatory	40-80 mg PO daily	Avoid if using LPV/r
pravastatin (Pravachol)	Off-label	Cardioprotection; immunomodulatory	80 mg PO daily	

References

1. Rationale for Consideration of Statins for COVID-19 Patients. Available online: https://www.massgeneral.org/assets/MGH/pdf/news/coronavirus/COVID-19_domID_statin.pdf (accessed on 4/10/2020; Verified 6/10/2020).
2. Marik, P. EVMS Critical Care COVID-19 Management Protocol. Available online: https://www.evms.edu/media/evms_public/departments/internal_medicine/EVMS_Critical_Care_COVID-19_Protocol.pdf (accessed on 5/8/2020).
3. Castiglione, V.; Chiriaco, M.; Emdin, M.; Taddei, S.; Vergaro, G. Statin therapy in COVID-19 infection. *Eur Heart J Cardiovasc Pharmacother* **2020**, doi:5826833 [pii] 10.1093/ehjcvp/pvaa042.
